# The Role of Non-Native Interactions in the Folding of Knotted Proteins: Insights from Molecular Dynamics Simulations

**DOI:** 10.3390/biom4010001

**Published:** 2013-12-24

**Authors:** Roberto Covino, Tatjana Škrbić, Silvio a Beccara, Pietro Faccioli, Cristian Micheletti

**Affiliations:** 1 Department of Physics, University of Trento, Via Sommarive 14, Trento 38123, Italy; E-Mail: faccioli@science.unitn.it; 2 INFN-TIFPA, Trento Institute for Fundamental Physics and Applications, Trento 38123, Italy; 3 SISSA—Scuola Internazionale Superiore di Studi Avanzati, Via Bonomea 265, Trieste 34136, Italy; E-Mails: tatjana.skrbic@pd.infn.it (T.Š.); michelet@sissa.it (C.M.); 4 Physics and Astronomy Department, University of Padua, Via Marzolo 8, Padova 35131, Italy; 5 Interdisciplinary Laboratory for Computational Science, FBK-CMM and University of Trento, Trento 38123, Italy; E-Mail: sabeccara@fbk.eu

**Keywords:** knotted proteins, folding simulations, non-native interactions

## Abstract

For several decades, the presence of knots in naturally-occurring proteins was largely ruled out *a priori* for its supposed incompatibility with the efficiency and robustness of folding processes. For this very same reason, the later discovery of several unrelated families of knotted proteins motivated researchers to look into the physico-chemical mechanisms governing the concerted sequence of folding steps leading to the consistent formation of the same knot type in the same protein location. Besides experiments, computational studies are providing considerable insight into these mechanisms. Here, we revisit a number of such recent investigations within a common conceptual and methodological framework. By considering studies employing protein models with different structural resolution (coarse-grained or atomistic) and various force fields (from pure native-centric to realistic atomistic ones), we focus on the role of native and non-native interactions. For various unrelated instances of knotted proteins, non-native interactions are shown to be very important for favoring the emergence of conformations primed for successful self-knotting events.

## 1. Introduction

Since the 1994 first survey of non-trivial entanglement in proteins [1], many instances of knotted and slipknotted proteins have been reported. In fact, a few hundred of the structures currently deposited in the Protein Data Bank (PDB) are known to contain knots [2,3,4,5,6,7,8,9,10,11] or, more precisely, *physical knots* (because a suitable arc closure of an open protein chain is needed to turn it into a ring with a mathematically well-defined topology [12,13]).

Regarding knots, naturally-occurring proteins differ in at least two major aspects with respect to flexible polymers of equivalent length. First, knots are statistically much rarer in proteins (arguably, for evolutionary reasons [14,15,16,17,18,19]). Secondly, the type, location and length of knots occurring in open flexible homopolymers have a stochastic character [20], whereas, for natively-knotted proteins, they are specific and robustly reproduced in repeated folding experiments [21] or unfolding/refolding cycles [22].

Important clues regarding the folding of knotted proteins have been recently provided by Mallam and Jackson for YibK and YbeA [21]. Specifically, they showed that these proteins, produced by a synthetic translational system, hence initially unknotted, can spontaneously fold to the native knotted state in the absence of any aiding cellular machinery (though the presence of chaperones can dramatically speed up the process). These observations support the notion that, as for proteins with non-entangled folding patterns [23,24], the intra-molecular interactions suffice to guide the folding process to the correctly knotted native state.

This is indeed a remarkable property if one considers that if the secondary and tertiary native contacts are progressively established in an arbitrary order, then it might be impossible to attain the correct topology. As a matter of fact, the very slow speed observed for the spontaneous folding reaction (10–20 min for YibK and YbeA [21]) is arguably indicative of a significant amount of backtracking from unproductive folding routes. This, in turn, is paralleled by the resilience to unknotting upon denaturation of the same molecule [9,21,25,26,27].

Computational approaches in several ways can complement the insight provided by experiments. In particular, over the past few years, various numerical studies have focused on elucidating the pathways connecting the unfolded and folded states of specific knotted proteins [14,15,19,28,29,30,31,32,33,34,35,36,37,38,39]. Because the typical folding timescale of knotted proteins is presently still beyond the reach of unbiased folding simulations, these studies were restricted, by necessity, to simplified or *ad hoc* folding contexts. In particular, most studies were performed with native-centric models, that is, in contexts where the native topology is favored by promoting the formation of native contacts. These approaches used lattice models of proteins [19,28,29], coarse-grained structural descriptions [14,30,31,33,34,35,36] and full atomistic ones [32]. Coarse-grained models were additionally used to investigate the role of non-native interactions [30,35]. Finally, realistic force fields were used in models with full atomistic details, where, moreover, either the folding dynamics was started from an ensemble of unfolded unknotted structures with a biasing molecular dynamics scheme or an unbiased molecular dynamics (MD) scheme was used from a selected repertoire of knotted and unknotted initial configurations [38,39].

**Figure 1:**
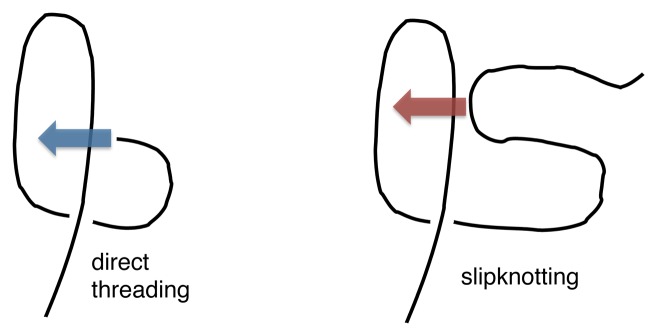
Sketch of common knotting mechanisms in polymers: direct threading and slipknotting.

These theoretical studies have been important for identifying and illustrating the most common mechanisms leading to knot formation. The two main ones are direct threading and slipknotting, as sketched in Figure 1. The former occurs when a loop is threaded by a straight protein terminus. In this case, the knot appears at one protein end and can then move or diffuse gradually towards the chain interior. The other common mechanism, first described in [22,33], is slipknotting, where a loop is threaded by a hooked or backward bent terminus. In this case, as soon as the bent end crosses the loop, the knot suddenly appears in the chain interior. The two mechanisms are very general and not specific to proteins, as was recently shown by studying the kinetics of spontaneous knotting and unknotting in flexible homopolymer chains [20].

Numerical investigations, besides profiling the possible modes of chain self-knotting that are *a priori* available to all proteins, can shed light on the physico-chemical mechanism that favors particular knotting pathways for specific proteins. One of the main emerging related issues regards the impact of native *versus* non-native interactions for the steering of the folding process towards the correct knotted state.

In fact, pure native-centric approaches have shown that native interactions alone are usually sufficient to drive proteins towards the knotted native geometry, mostly via a slipknotting mechanism. On the other hand, starting from the work of Wallin *et al.* on the trefoil-knotted YibK [30], it has been known that the folding efficiency in coarse-grained models of knotted proteins can be dramatically enhanced by specific sets of non-native interactions [30,35,38].

Motivated by these earlier studies, we have taken advantage of recent theoretical and computational developments in advanced sampling techniques of productive folding pathways and used them to characterize the detailed folding pathway of a short trefoil-knotted protein using a realistic atomistic force field. Specifically, we considered MJ0366 and studied it with full atomistic resolution and with the AMBER99SB-ILDN [40] implicit solvent force field, using a dominant reaction pathway (DRP) sampling scheme to assess the statistical significance of productive folding trajectories obtained with ratchet-and-pawl molecular dynamics simulations.

Here, we review and revisit the results obtained with this approach and compare them, within a common interpretative framework, with results from Monte Carlo (MC) simulations based on coarsegrained models using both purely native and native-centric potentials. From the analysis, it emerges that, for at least two knotted proteins, non-native interactions arguably play a crucial role in steering the formation of the native topology and in establishing the weight of specific pathways.

The paper is organized as follows. First, we briefly present the DRP approach and illustrate its effectiveness and reliability for capturing the detailed mechanisms of the folding process of unknotted proteins. We next move to consider the case of a small knotted protein. We then investigate the folding of two other evolutionarily related proteins, one of which displays a knot that is missing in the other.

## 2. Validation: Folding a WW Domain

The dominant reaction pathways (DRP) approach was designed to efficiently obtain the most likely pathways between a given denatured configuration and the native state [41,42,43].

The folding process is described with a discrete over-damped Langevin dynamical evolution, which is formally written as:

(1) $${\text{x}}_{i+1}={\text{x}}_{i}+\frac{\Delta tD}{k_{B}T}F({\text{x}}_{i})+\sqrt{2D\Delta t}\eta_{i}$$

where the 3*n*-dimensional vector, x, describes the position in space of the *n* particles in the system and **F** is the corresponding 3*n*-dimensional force vector. In coarse-grained contexts, we represent each amino acid with a single C*_α_* centroid; in this case, *n* is equal to the number of protein amino acids. In atomistic contexts, instead, *n* is equal to the number of atoms in the protein. *i* = 1,…,*N* is an index labeling time steps, *D* the diffusion constant, *k_B_* the Boltzmann constant, *T* is the heat-bath temperature, Δ*t* the integration time step and *η_i_* a white Gaussian noise with unitary variance.

It is known that, in the stochastic dynamics defined by Equation (1), the probability density of each productive trajectory is proportional to the negative exponential of its Onsager–Machlup action [44], which can be written as:

(2) $$S_{OM}=\frac{m\gamma }{4k_{B}T\Delta t}{\sum_{i=1}^{N}\left({\text{x}}_{i+1}-{\text{x}}_{i}-\text{F}(x_{i})\frac{\Delta t}{\gamma }\right)}^{2}$$

where γ is the friction coefficient.

The Onsager–Machlup action is thus the scoring function in the DRP approach: within a trial set of trajectories, the path with lowest Onsager–Machlup action is the one with the highest probability to be realized [41,45,46].

A sizeable number of reactive trajectories is produced in parallel by means of a biased MD algorithm, called ratchet-and-pawl molecular dynamics (rMD) [47]. The rMD computational cost is far smaller than that of a regular MD simulation, although the amount of work done by the biasing force is kept at a minimum. In fact, the system is let to spontaneously evolve whenever the unbiased dynamics leads to increasing of the fraction of native contacts. On the contrary, a bias potential makes it unlikely that the system visits again configurations sharing a lower similarity with the native one. In this way, thermal fluctuations drive the system towards the native state. The bias potential assists the system in overcoming free energy barriers, exerting its action along the fraction of native contacts, which has proven to be a reasonable reaction coordinate for the folding protein process [48,49]. With reference to Equation (2), the *unbiased* probability is calculated, by including in the Onsager–Machlup action only the unbiased force, given by the negative of the gradient of the molecular potential energy. Finally, one has to select the trajectory characterized by the lowest action, i.e., the highest probability according to the *unbiased* dynamics and, thus, the least biased one.

**Figure 2:**
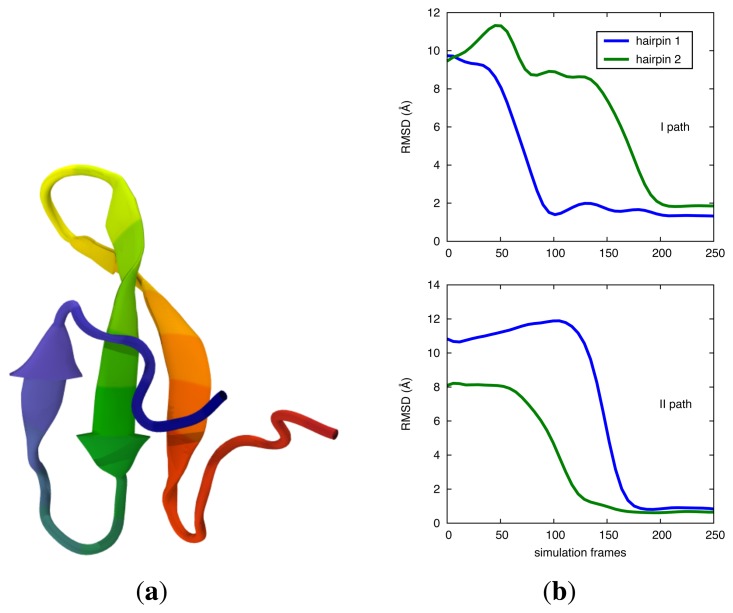
(**a**) Cartoon of the native conformation of fip35 WWdomain (Protein Data Bank (PDB) entry: pin1). Here, and in subsequent protein representations, a rainbow coloring scheme is used to color the protein chain from the N terminus (red) to the C one (blue). (**b**) Two examples of the possible folding pathways found in DRP atomistic folding simulations. The progress to the native state is captured by computing the root mean square distance (RMSD) relative to the native coordinates respectively for the first and second *β*-hairpins. In the upper panel, it can be seen that the first hairpin (in blue) folds first, followed by the second hairpin (green). The lower panel shows the other mechanism, in which the order of hairpin formation is reversed. Adapted with permission from [50].

To validate the DRP approach, we first studied the folding of Fip35, a small WWdomain essentially comprised of two beta-hairpins (see Figure 2a) [50]. This domain has been studied experimentally, and its folding time is the shortest known for a WW domain, at about 13 μs. Results are available also from MD simulations performed on the special-purpose computer, ANTON [51].

Using the AMBER99SB-ILDN fully atomistic force field [40] with implicit solvent (generalized Born) [52], we simulated the folding process starting from 44 different initial configurations, generated by thermal unfolding, and identified the most probable among 48 independent trial trajectories for each initial configuration.

Besides characterizing the folding dynamics with the DRP scheme, we also used a Monte Carlo approach to profile the protein free-energy landscape. In this case, we resorted to coarse-grained models based on the sole C*_α_*-centroid amino acid representation and considered two very different force fields: one incorporating only native-centric (Gō) interactions [53] and another one with *additional* accounts for non-native interactions via quasi-chemical potentials. The latter, in fact, capture the normalized statistical propensities of the different amino-acid pairs to be in contact in a protein's native states [54].

**Figure 3:**
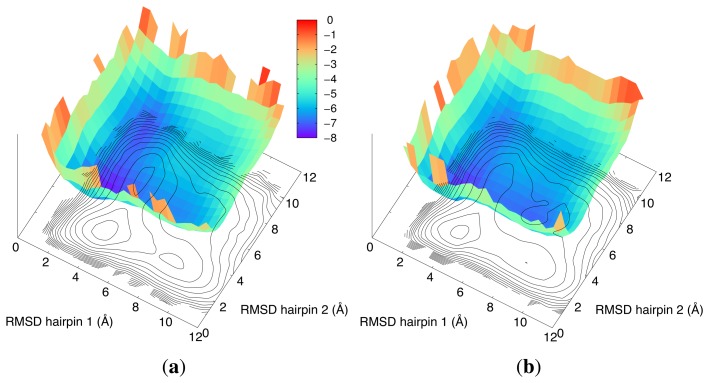
Free-energy landscape of fip35 computed in the two-dimensional coordinate space defined by the RMSD relative to the native coordinates of the respective hairpins. Contour lines are separated by a change of 0.8 kcal/mol in the free energy. The same scale is used for the color panel. The data in panel (**a**) pertain to Monte Carlo folding simulations employing a purely native-centric coarse-grained model; the data in panel (**b**) incorporate, in addition, non-native quasi-chemical interactions. Adapted with permission from [50].

In Figure 2b, we illustrate the two possible folding pathways obtained by atomistic DRP simulations, which differ by the order of formation of the two hairpins. These results are in agreement with millisecond-long unbiased MD simulations [51,55,56]. The two-pathway picture also makes it possible to quantitatively explain the existing experimental *ϕ*-values [57,58]. The MC simulations shown in the two panels of Figure 3 give qualitatively similar results.

As for the relative probabilities of the two folding pathways, we estimated the relative reaction rate by means of a Kramers approach. To evaluate the free energy difference in the transition state, we employed Monte Carlo simulations in the coarse-grained force field. The probabilities we thus obtained are 70% *versus* 30%, in good agreement with the experimental results analyzed by Weikl [58], on *ϕ*-value analysis data. Moreover, an MC integration at 380 K gives a probability for pathway 1 of about 60%, in qualitative agreement with available experimental results [57,58]. Furthermore, the ANTON frequencies, even though estimated on a limited sample, compare fairly well, giving an 80 to 20 ratio [55].

The comparison of our atomistic DRP results on fip35 with coarse-grained free energy plots obtained by MC and with experimental and computational results obtained by other groups allowed us to validate the DRP approach. The number and hierarchical structure of the dominant pathways and their relative probability are correctly predicted by our simulations, based on a statistically significant set of trajectories.

In addition, the results shown in Figure 3 allow us to discuss if, and to what extent, the folding process of fip35 is influenced by the interplay of native and non-native interactions. Over the years, this classic question has been addressed with various methods, including computational approaches of increasing detail and sophistication. These studies clarified that non-native interactions can contribute as much as native ones to a protein's internal energy [59,60], although their impact on the folding process can vary significantly from case to case.

On the one hand, in fact, studies based on lattice and off-lattice models have pointed out the importance of non-native interactions in increasing the folding efficiency. In particular, it was shown that small (random or attractive) non-native perturbations of the energy surface can effectively decrease the folding free-energy barrier and, hence, accelerate the folding kinetics [61,62]. In addition, by using a lattice model, it was shown that specific non-native interactions can favor the “climbing” of the free-energy barrier, by assisting the formation of a folding nucleus [63].

On the other hand, several studies argued that non-native interactions play a minor role in shaping the folding mechanism, suggesting that the ideal native-centric picture is a good representation of realistic folding reactions. In particular, Gin and co-workers observed that the order of native-contact formation is not significantly influenced by the inclusion of non-native interactions [64]. More recently, Best and co-workers used the results of extensive molecular dynamics simulations performed on the ANTON supercomputer to perform a comparative analysis of the frequency of formation and the duration of native and non-native contacts for several small globular proteins [65]. They observed that the type, lifetime and frequency of non-native contacts, which are formed in the unfolded state, are statistically consistent with those which are found to form during the folding reaction. The finding leads one to argue that non-native interactions do not play a specific role in promoting the folding, and they mostly provide an overall effective friction.

These two sets of conclusions are not necessarily mutually exclusive. Indeed, a possible scenario is one in which the folding mechanism is mostly shaped by the native interactions, while the fine-structure of the landscape, such as the height of the free-energy barriers, is significantly influenced by non-native effects.

The results obtained in the different CGsimulations reported in the two panels of Figure 3 are compatible with this hypothesis, by showing that inclusion of non-native interactions do not significantly modify the two-pathway folding mechanism of fip35, but only leads to a slight reduction of the free-energy differences, which are encountered along the folding pathways.

It is important to stress that the considerations made so far regard the case of unknotted proteins. The relative importance of native and non-native interactions in driving the folding reaction may depend, in a critical way, on the presence of topological constraints, such as, e.g., those provided by an external membrane to which the protein is tethered [66], by the presence of disulfide bonds [67,68,69,70] or of knots in the native structure.

Hence, the question naturally arises as to whether the same theoretical conclusions remain valid also in the presence of knots, which clearly unbalance the energy-entropy competition. This point is discussed in the next sections by considering the folding of knotted proteins.

## 3. Folding of a Small Knotted Protein

**Figure 4:**
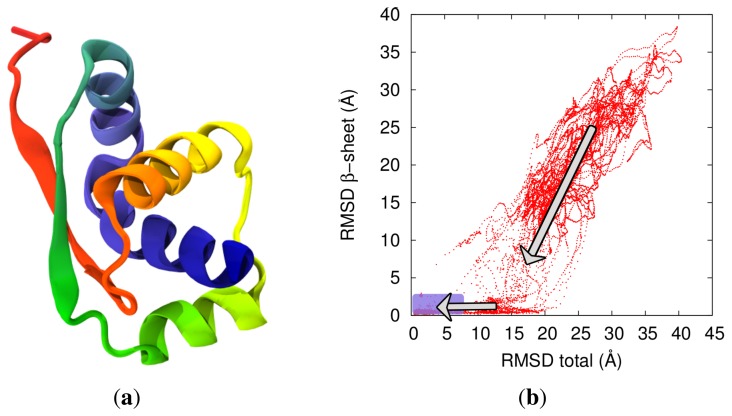
(**a**) Cartoon of the native conformation of MJ0366 (PDB entry: 2efv). The *β*-sheet locks the loop that is threaded by the *C*-terminus in the native fold, thus producing a trefoil knot. (**b**) Atomistic DRP folding trajectories projected on the plane defined by the RMSD relative to the native coordinates of the non-local *β*-sheet and the RMSD relative to the native coordinates of the total structure. Arrows indicate the directionality of the folding trajectories. It is noticed that the *β*-sheet has to be well-formed before the trajectories proceed to the native knotted state. The purple overlayed rectangle highlights the region encompassing all first knotting events. Adapted with permission from [38].

The computational strategies validated on the small WW domain were then applied to the 82 residue-long MJ0366 protein (PDB entry: 2efv), a natural homo-dimer and the smallest known knotted protein. The monomeric unit of MJ0366 displays a trefoil knot at the *C*-terminus. The knot is rather shallow, in that about six amino acids of the *C*-terminal helix protrude from the native loop; see Figure 4a.

The folding mechanism of MJ0366 has not yet been experimentally characterized, but it has been numerically investigated. The first and seminal numerical study of MJ0366 was carried out by Noel *et al.* [32], in the context of a protein model with atomistic resolution, but a simplified native-centric force field. By carrying out extensive folding simulations, it was shown that promoting the formation of native contacts alone was sufficient to fold the protein to the knotted native configuration. Furthermore, it was shown that slipknotting was statistically much favored over direct threading. Interestingly, the relative statistical weight of these two knotting mechanisms was found to depend strongly on the nominal temperature of the folding simulations [32]. This fact is arguably indicative that the knotting pathways are sensitive to a delicate enthalpic-entropic balance and calls for the necessity of using more refined potentials to ascertain the impact of non-native interactions on the folding process of MJ0366.

This observation motivated us to use the combination of ratchet-and-pawl MD simulations and DRP schemes to investigate the folding pathways of MJ0366 with the AMBER99SB-ILDN force field. To our best knowledge, this represented the first endeavor to systematically map the folding and self-entanglement process of a knotted protein using a realistic force field, albeit in implicit solvent.

To ensure a broad coverage of the possible starting conditions for the folding trajectories, we first generated 100 different unfolded configurations by MD runs at high temperature, all starting from the native state. We checked that all unfolded configurations, which are typically extended, were unknotted. Next, we evolved each one of these initial states with 40–50 independent rMD simulations. As already mentioned, in the rMD scheme, the system is allowed to evolve freely towards configurations with increasing overall geometrical similarity with the native state, but it is prevented from regressing to conformations with lower similarity.

It should be noted that the rMD bias is different compared to standard steered-MD approaches, which drive the system regardless of whether it progresses or regresses along the reaction coordinate. Furthermore, the fact that the latter is based on a measure of an overall similarity has the advantage of not favoring specific sets of native or non-native contacts nor their order of formation.

As a matter of fact, only a few percent of the 4,000 simulated rMD trajectories reached the correctly knotted native state. To further minimize the effects of the rMD bias, we finally retained only one productive folding trajectory per initial state, namely the one with the highest statistical weight according to the DRP criterion.

An informative overview of the salient steps that lead to the native knotting across the selected trajectories is shown in Figure 4b. The figure illustrates the selected trajectories projected on the plane defined by the root mean square distance (RMSD) relative to the native coordinates of the whole molecule and the RMSD relative to the native coordinates of the *β*-sheet. These two parameters are useful in that they allow us to simultaneously monitor the overall progress towards the native state, the formation of non-local *β*-sheet and the onset of knotting.

The data in Figure 4b clarify that the native knotted topology is typically established after the formation of the *β*-sheet and when the overall geometrical similarity is already high. As a matter of fact, we observed that at the time of the first knotting event, 80% to 95% of the native contacts are already established. This is indicative of a rather late stage of the formation of the native knot.

**Figure 5:**
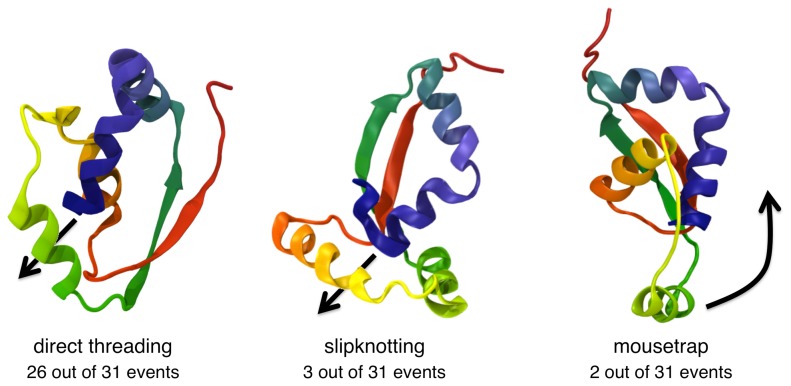
Schematic representation of the observed knotting mechanisms for MJ0366. The largely dominant mechanism is direct threading, when a straight, helically-arranged *C*-terminus threads an already formed native loop, locked by the non-local *β*-sheet. Occasionally, the knot can form via slipknotting. This happens when the loop is threaded by a backward-bent *C*-terminus. A further, though rare, knotting mechanism is mousetrapping, which arises when the loop region concertedly rotates and entraps the *C*-terminus. Adapted with permission from [38].

The late formation of the knot is consistent with what was observed in [32,36] on the basis of a pure native-centric model for MJ0366 folding. The two approaches, however, lead to very different conclusions regarding the dominant knotting mechanism. Our simulations, instead, indicate that direct threading is much more common than slipknotting. Indeed, as indicated in Figure 5, for 26 of the 31 considered productive trajectories, the knot is established when the *β*-loop is directly threaded by the *C*-terminus in a non-bent geometry (because the prior formation of the terminal native helix prevents the C terminus from bending appreciably). Slipknotting events, associated with the threading of a bent *C*-terminus, was observed for only three of the 31 trajectories.

Interestingly, an additional and previously unreported knotting mode was also observed and was termed the mouse-trapping mechanism, as it involves the concerted motion of the *β*-loop over the C terminus, as illustrated in Figure 5. This mechanism is observed in only two of the 31 considered trajectories.

Because both approaches describe MJ0366 on the basis of a fully atomistic force field and differ only in the interactions considered (purely-native centric vs. AMBER99SB-ILDN), it is plausible to conjecture that the qualitatively different dominant knotting modes originated from the different treatment of non-native interactions.

To check this hypothesis in the most transparent way, we turned to the coarse-grained model introduced in the previous section, where the interplay of native- and non-native interactions can be conveniently varied [38]. In order to investigate the folding mechanism of these proteins within an affordable computational time, we adopted a Monte Carlo stochastic evolution, which, in view of the locality of the employed moves (local crankshafts and single-bead displacements), can be regarded as a proxy for actual overdamped polymer dynamics (see, e.g., [71]).

It is useful to recall that for the unknotted small WW domain, the coarse-grained folding trajectories were largely insensitive to whether the force field accounted for native-only interactions or for non-native ones, too; see Figure 3. However, this is not the case for the knotted MJ0366 protein.

Specifically, non-native interactions greatly enhance the probability of attaining the correct native knotted configuration. In fact, in a set of thousands of equilibrated coarse-grained configurations of MJ0366 generated via Monte Carlo, it was observed that the knotting probability was only 75% when non-native interactions were accounted for and only 12% when they were disregarded.

A good consistency with the atomistic folding trajectories was observed regarding two general aspects of the MJ0366 knotting process. First, in productive coarse-grained trajectories, the knot is established at a rather late stage. Secondly, the dominant knotting mechanism is again found to be the direct threading of the native loop locked by the non-local *β*-sheet. The presence of the sole native interactions lead to a frequent slipknotting mechanism (observed in eight out of ten productive trajectories). The scenario completely changed considering also non-native interaction, since in all 10 productive trajectories, knotting formation occurred via direct threading. The analysis and inspection of the Monte Carlo folding trajectories indicated that non-native interactions promote an overall attraction of residues in the two beta-strands, which reinforces the native propensity of *β*-sheet formation and, hence, appears instrumental for ensuring that it is formed earlier in the folding process than when native-only interactions are considered.

The results of atomistic and coarse-grained simulations provide a convergent indication that non-native interactions can efficiently drive the knotting process of MJ0366. The analysis of the successful and unsuccessful knotting event in different models with native and non-native interactions suggests that the latter aid knotting by favoring a specific temporal succession of secondary and tertiary contacts: first, the formation of the *C*-terminal helix and the *β*-loop and, next, the (direct) threading events. A specific illustration of an unsuccessful knotting event due to an unfavorable sequence of contact formation is given in Figure 7 of [38].

The latter mechanism, which is supported by the calculation of the effective loop; *C*-terminus interactions within the atomistic trajectories, are reinforced in the very recent investigation of [39]. In this study, Noel *et al.* extended their earlier atomistic study of MJ0366 by taking it from native-centric only to a realistic atomistic force field.

It is particularly interesting to compare the previously discussed findings with the ones of [39], because the two studies rely on different, and complementary, methodological choices for making the computational cost of the folding simulations manageable. In fact, in our earlier study [38], a large ensemble of fully unfolded trajectories is used to follow the folding dynamics using the rMD biasing and DRP reweighting schemes with a realistic, though implicit solvent, force field. In the later study of Noel *et al.*, the folding dynamics simulations are unbiased and based on explicit treatment of the solvent effects, but the starting configurations are selected *a priori* to be primed for *C*-terminal slipknotting. It should be also mentioned that in their studies, Noel and co-workers considered a slightly longer version of protein MH0366 than the one adopted in our DRP simulations reported in [38]. In fact, Noel *et al.* also accounted for a few additional residues at the *C*-terminus that are unresolved in the PDB structure used by a Beccara *et al.* It therefore cannot be ruled out *a priori* that the entropic effects associated with the longer terminus can enhance the occurrence of the slipknotting.

Consistently with what was seen in [38], the findings of [39] confirm the relevance of non-native interactions in the folding of MJ0366. In particular, by examining in detail the unbiased MD trajectories, it is suggested that a crucial interaction is the transient formation of non-native salt bridges, playing a key role in the advancement of the C-terminus through the loop.

In the future, it would be interesting to compare within the same simulation scheme the folding pathways of protein MJ0366 with that of an unknotted synthetic counterpart, obtained, e.g., from homology modeling. Such comparative studies have been previously undertaken in other contexts, as discussed in the next section, where we compare the knot-promoting role of non-native interactions in two evolutionarily related proteins, whose native structures differ mostly by the presence or absence of a knot.

## 4. Knotting in Larger Proteins: Transcarbamylases

The phenomenology presented for the WW domain and MJ0366 provides a vivid illustration of the very different degree of coordination of the various folding steps required to attain the native state depending on its unknotted or knotted character. This, in turn, reverberates on a different role and impact of non-native interactions. Based on the simulated trajectories, they have a negligible impact on the folding of the unknotted WW domain (which, in fact, can proceed through multiple pathways involving a very different order of formation and assembly of secondary structure elements). On the other hand, they appear to be very important for priming the knot formation in MJ0366 by drawing the C-terminus close to the *β*-loop.

While the involvement of non-native interactions is not *a priori* necessary for the folding of knotted proteins (see, e.g., [32,33]), it is interesting to investigate how common this mechanism can be.

In this respect, it is important to recall the seminal study of Wallin *et al.* [30], who first investigated the impact of specific non-native interactions on YibK and concluded in favor of their relevance to enhance the speed and yield of the YibK folding process.

**Figure 6:**
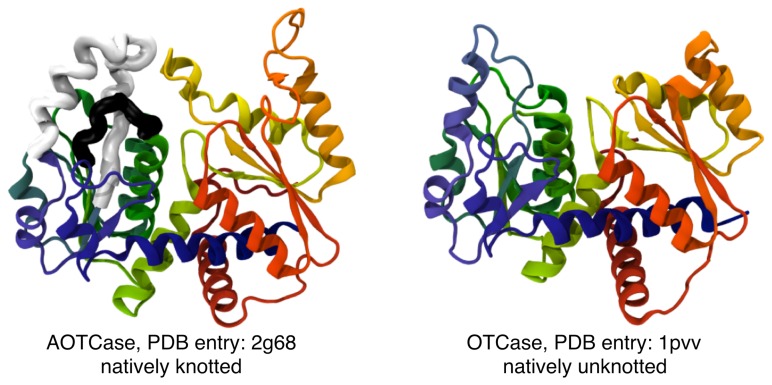
Knotted and unknotted carbamoyltransferases. Cartoon representation of AOTCase (on the **left**), which is natively-knotted in a right- handed trefoil knot, and the unknotted OTCase (on the **right**). The two proteins have a noticeably-good structural correspondence. A key difference is the presence of the two “knot-promoting” loops [8] in the AOTCase (thick black and white segments). The virtual bridging of either of both of these loops unknots the AOTCase structure. Adapted with permission from [35].

Further molecules that display the same effects are two homologous carbamoyltransferases: the N-acetylornithine carbamoyltransferase (AOTCase, PDB entry: 2g68) and ornithine carbamoyltransferase (OTCase, PDB entry: 1pvv). The overall geometrical similarity of the two proteins is very significant (*p*-value = 10^−6^ according to the Mistral structural alignment server [72]), as visible in Figure 6. Yet, their knotted state is different: OTCase is unknotted, while AOTCase is trefoil-knotted [4,8,9]. As in other notable instances of structurally-related knotted-unknotted protein pairs [8], the different topology of the two proteins is associated with the presence of one or more extra loops in the knotted variant.

As detailed in [35], the early folding kinetics of each of these two proteins was studied using the same coarse-grained scheme and MC algorithm described in the previous sections, namely by first promoting only native interactions, and then by additionally including non-native interactions, too.

This significant sequence and structural similarity of the two proteins is ideally suited to carry out a comparative study of the impact of non-native interactions on their folding process. In addition, both molecules are significantly longer than the 82residue-long MJ0366. In fact, the AOTCase and OTCase consist of 332 and 312 amino-acids, respectively. For this reason, the unbiased, coarse-grained (C*_α_*-trace) folding simulations were followed not to completion, but up to a stage where, on average, 35% of the native contacts are established [35].

At this folding stage, the amount of native secondary structures varies greatly, depending on contact locality: only ∼15% of native contacts involving *β* strands are present, while about 55% of the *α*-helical native contacts are formed. In particular, the *C*-terminal *α*-helix was typically fully formed.

For both transcarbamylases, the folding kinetics was characterized with more than one hundred simulations performed with two different force fields: pure native-centric and native-centric with added non-native quasi-chemical interactions (for a detailed description of the model, see [73]). This comparative strategy allowed us to address two questions: (i) whether the natively-knotted ATOCase and the natively-unknotted OTCase displayed any different propensity to self-entangle in the early folding stages and (ii) whether non-native interactions play any major role in it.

**Figure 7:**
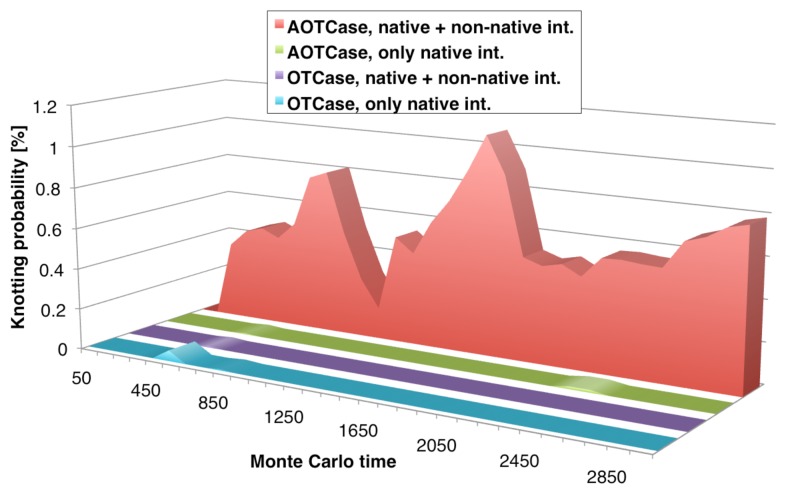
Monte Carlo time-evolution of the average knotting probabilities of the natively-knotted AOTCase and of the unknotted OTCase starting from unfolded conformations up to a stage when about 35% of the native contacts are formed. For each protein, it the average knotting probability is shown as observed when using a purely native-centric force field and with the addition of non-native quasichemical interactions. Adapted with permission from [35].

The time-evolution of the average knotting propensity for the coarse-grained models of AOTCase and OTCase and its dependence on native and non-native contacts is strikingly illustrated in Figure 7.

Two conclusions can be drawn from the data shown. First, the incidence of knots in the natively-unknotted OTCase simulations remains negligible at all times and regardless of the force field. Secondly, the knotting propensity of AOTCase is dramatically enhanced by introducing non-native interactions. This result parallels the findings for MJ0366 and provides a further illustration of the importance that non-native interactions can have for enhancing the efficiency of knot formation in natively-knotted proteins.

Because the knot-promoting role of non-native interactions is expected to vary across proteins (for instance, in OTCase, non-native interactions do not promote knotting; see Figure 7), it is interesting to discuss in more detail the general characteristics of AOTCase knotting.

In this respect, we first mention that the knots formed in the folding simulations of AOTCase are all trefoils, as is the native AOTCase knot. Their handedness, or chirality, is also equal to the native one.

Next, it should be noted that the observed knots are not irreversible. In fact, though AOTCase knots are long-lived (their individual lifetime could be up to one-tenth of the total folding simulations), they can untie, similarly to the spontaneous knotting and unknotting recently characterized for flexible chains. At variance with the latter case, however, AOTCase knotting does not occur at both protein termini. In fact, all knotting events resulted from the threading of the fully-folded *C*-terminal *α*-helix through various pre-formed protein loops. The latter typically spanned a few tens of residues located in various regions of the chain (mostly regions with indices 55–65, from 90–110 and from 125–155). Because of the variability of the threaded loop, the protein region accommodating the reversible trefoil knots did not show any substantial overlap with the native knotted region.

Finally, by investigating the systematic differences in the pattern of contacts established by the *C*-terminus of AOTCase and OTCase, it was found that the overall strength of the non-native attraction is consistently stronger (by about 15%) in the case of the natively-knotted AOTCase than for OTCase. It should be considered, in fact, that this average is obtained considering all sampled structures, irrespective of their degree of compactness and self-entanglement (knotted) state. On the other hand, the analysis did not indicate any major enthalpic effect favoring the interaction of the *C*-terminus and specific AOTCase regions, such as loops.

In summary, the findings support a scenario where non-native interactions favor the self-entanglement of AOTCase through a general attraction of the *C*-terminal *α*-helix toward the rest of the partially-folded chain, which can hence favor loop threading events. In contrast, threading events in OTCase are disfavored by the fact that the *C*-terminal helix typically points away from the partially folded globule rather than being attracted towards it.

Along with what is presented for MJ0366, these results emphasize that the self-tying of a polypeptide chain implies that native contacts, or, at least, sets of native contacts, have to form in a specific order. Consequently, in a perfectly smooth landscape, most of the reaction pathways that would lead to the folding of a topologically trivial protein will be unproductive for a knotted protein. In this case, the chain will have to backtrack many times to an unfolded configuration before it can find a correct folding pathway and reach the native state. One may therefore expect that the folding of knotted proteins may not be well described within the framework of the minimal frustration principle, i.e., that the small sequence-dependent corrugations of the energy landscape do not simply provide an overall “democratic” effective friction allowing the co-existence of a large number of nearly equiprobable pathways, but, rather, are crucial to guide the chain through the relatively few routes that can lead to the knotting of the chain.

## 5. Conclusions

Much of the growing interest in knotted proteins originates from the challenges posed by the attempt to understand the general mechanisms that allow these molecules to overcome or minimize the several kinetic or topological bottlenecks and free-energy barriers that line the folding routes to a knotted native state.

In this endeavor, computational approaches can appropriately complement the insight offered by experimental investigations. In particular, by using and comparing models of increasing detail and sophistication, it is possible to formulate a hypothesis on the physico-chemical mechanisms that are arguably responsible for guaranteeing the efficiency of the folding process by favoring the correct order of the formation of secondary and tertiary structures.

Here, we revisited, within a unified conceptual framework, a number of such computational studies that have been carried out in recent years. In particular, the analysis has been largely directed towards discussing the role of native and non-native interactions in knotted proteins.

The role of non-native contacts in guiding the knot formation was first emphasized by Wallin *et al.* [30], who investigated the folding of the knotted protein, YibK, using a simplified native-centric model, and observed that the introduction of *ad hoc* non-native interactions could dramatically enhance the knotting propensity, which were otherwise negligible in pure native-centric contexts.

The cases discussed here extend and generalize these studies and correspond to a set of knotted proteins (namely MJ0366 and an AOTCase) and unknotted terms of reference for which the full or partial folding process was characterized using models with different structural resolution (coarse-grained or atomistic) and with different force fields (native-centric with and without quasichemical non-native interactions, or with realistic atomistic interactions with and without explicit solvent).

Notwithstanding the differences in structural detail and force fields, the evidence gathered here provides a very consistent indication that non-native interactions are very important for promoting the emergence of protein conformations which are primed for establishing the native knotted topology. In both considered cases, the mechanism appears to involve an effective attraction of the *C*-terminus to a specific loop that, once threaded, leads to the native knotted state. It is interesting to observe that the same general mechanism was argued to be optimal for enhancing the knotting probability of YibK [30].

While there is no *a priori* reason for non-native interactions being necessarily crucial for the folding of knotted proteins in general [32,33], it is noteworthy that folding simulations of three unrelated knotted proteins consistently point to the relevance of this mechanism.

Accordingly, it would be most interesting to extend the analysis of the impact of non-native interactions to the folding of other knotted proteins. The few pairs of knotted-unknotted proteins with significant overall structural similarities ought to be ideally suited for these future investigations [8].
